# Interleukin-17 regulation: an attractive therapeutic approach for asthma

**DOI:** 10.1186/1465-9921-11-78

**Published:** 2010-06-16

**Authors:** Seoung Ju Park, Yong Chul Lee

**Affiliations:** 1 Department of Internal Medicine and Research Center for Pulmonary Disorders, Chonbuk National University Medical School, Jeonju, South Korea

## Abstract

Interleukin (IL)-17 is recognized to play a critical role in numerous immune and inflammatory responses by regulating the expression of various inflammatory mediators, which include cytokines, chemokines, and adhesion molecules. There is growing evidence that IL-17 is involved in the pathogenesis of asthma. IL-17 orchestrates the neutrophilic influx into the airways and also enhances T-helper 2 (Th2) cell-mediated eosinophilic airway inflammation in asthma. Recent studies have demonstrated that not only inhibitor of IL-17 per se but also diverse regulators of IL-17 expression reduce antigen-induced airway inflammation, bronchial hyperresponsiveness, and Th2 cytokine levels in animal models of asthma. This review will summarize the role of IL-17 in the context of allergic airway inflammation and discuss the therapeutic potential of various strategies targeting IL-17 for asthma.

## Introduction

Asthma is a common airway disorder that is characterized by chronic airway inflammation, mucus production, and airway hyperresponsiveness (AHR) with airway remodeling. Airway inflammation in asthma usually involves polarization of the T lymphocyte response to T-helper 2 (Th2) cells [1]. The pathologic role of Th2 cells is mediated through the release of Th2 cytokines that are essential for immunoglobulin E (IgE) synthesis, chemokine production, airway eosinophilia, smooth muscle hyperplasia, mucus production, and AHR [2-4]. As Th1 cells secrete interferon (IFN)-γ that inhibits the proliferation of Th2 cells, Th1 cells have been suggested to display a regulatory function in allergic asthma [4]. Thus, the concept of Th1/Th2 paradigm has been of vital interest to grasp the molecular and cellular mechanism and discover therapeutic modalities in asthma. Recently, a third subset of effector helper T cells that exhibit functions distinct from Th1 and Th2 cells and preferentially produce interleukin (IL)-17 (named Th17 cells) has been discovered, updating the Th1/Th2 paradigm [5-7]. On allergen sensitization, Th17 cells home to the lung and enhance not only neutrophilic airway inflammation but also Th2 cell-mediated eosinophilic airway inflammation in mouse models of asthma [8,9]. These observations have indicated that investigation of the differentiation, effector function, and regulation of Th17 cells may offer a new way to control asthma.

The IL-17 family consists of six members including IL-17 (now synonymous with IL-17A), IL-17B, IL-17C, IL-17D, IL-17E (also called IL-25), and IL-17F [10]. IL-17, the most investigated member in this family, exerts a wide variety of biological activities due to ubiquitous distribution of its receptor [10]. IL-17 is implicated in numerous immune and inflammatory responses primarily as a pro-inflammatory regulator by inducing the expression of various inflammatory mediators, such as cytokines, chemokines, adhesion molecules, and growth factors [6,11-13]. There is emerging evidence that an increase in IL-17 level is closely associated with a range of inflammatory diseases including rheumatoid arthritis, multiple sclerosis, inflammatory bowel diseases, and psoriasis [14,15]. In asthmatic patients, IL-17 expression has been shown to increase in sputum, lung cells, bronchoalveolar lavage (BAL) fluids, and peripheral blood [16-21]. Evidence for the involvement of IL-17 in the pathogenesis of asthma is further provided by the finding that expression of IL-17 mRNA is up-regulated in the airways of a mouse model of asthma [18]. Therefore, IL-17 has been suggested as a crucial regulator of allergic asthma. In this review, we focus primarily on the regulatory pathways and roles of IL-17 in airway inflammation and scrutinize the therapeutic potential of various strategies targeting IL-17 for asthma.

### IL-17: sources and regulation of production

IL-17 was identified as a rodent complementary DNA transcript named cytotoxic T-lymphocyte-associated antigen 8 (CTLA-8) in 1993 [19]. Originally, CTLA-8 was not recognized as a cytokine due to its unusual amino acid sequence. However, subsequent characterization revealed that this molecule is produced by T cells and thus renamed as IL-17 [20,21]. Genomic sequencing led to the discovery of five additional family members designated IL-17B, IL-17C, IL-17D, IL-17E, and IL-17F [10]. Even though the cellular sources and expression patterns of the mammalian IL-17 family members are different, they all exert pro-inflammatory activity [22]. Among the IL-17 family members, the most investigated cytokine is IL-17. IL-17 is a disulfide-linked homodimeric glycoprotein consisting of 155 amino acids with a molecular weight of 35 kDa [20,23]. It has been known that IL-17 is produced predominantly by a specific subset of Th cells, namely Th17 cells [5]. Additionally, other cell types such as CD8^+ ^T cells, γδ T cells, and natural killer T cells also produce IL-17 [14,24,25]. Eosinophils, neutrophils, macrophages, and monocytes can also be sources of IL-17 in some cases [7,16,23].

The differentiation of Th17 cells from naïve T cells depends on the combination of IL-6 plus transforming growth factor (TGF)-β [26,27]. In the presence of IL-6 and TGF-β, a specific Th17 cell transcription factor, retinoic acid receptor-related orphan receptor (ROR)-γt is up-regulated [28]. While IFN-γ and IL-4 produced by Th1 and Th2 cells, respectively, are able to reinforce the differentiation to polarized T cell subtype acting as an autocrine factor, IL-17 does not enforce the differentiation of Th17 cells [26]. Instead, IL-21 produced by Th17 cells acts in a positive feedback loop to differentiate Th17 cells [29]. IL-23 expands and stabilizes Th17 cells to produce IL-17, IL-17F, IL-21, and IL-22 [30,31]. In addition, signal transducer and activator of transcription 3 (STAT3) appears to be the essential signaling molecule for the differentiation of Th17 cells because IL-21 is induced in a STAT3-dependent manner [32,33]. Recent studies with both human and mouse have demonstrated that IL-1β is essential in the early differentiation of Th17 cells and conversion of Foxp3^+ ^T cells into IL-17-producing cells [34,35]. IL-1β synergizes with IL-23 and IL-6 to regulate Th17 cell differentiation and to maintain cytokine expression in effector Th17 cells [34]. Altogether, there is a variety of molecules regulating the differentiation or stabilization of Th17 cells, and they can be attractive targets for blocking IL-17 generation.

### IL-17: signaling pathway and biological roles

A first receptor for IL-17, IL-17R (renamed IL-17RA), was described with the discovery of IL-17 [19,20]. Similar to the IL-17 cytokine family, IL-17 receptors form a unique family composed of five members which are IL-17RA, IL-17RB, IL-17RC, IL-17RD, and IL-17RE [22]. Among these IL-17 receptors, IL-17RA is the cognate receptor for IL-17, and human IL-17RC also binds IL-17 in spite of its higher affinity for human IL-17F [36]. Toy *et al *have demonstrated that the biological activity of IL-17 is dependent on a formation of receptor complex composed of IL-17RA and IL-17RC, providing a potential framework for elucidating the interactions between the expanded family of IL-17 ligands and their receptors [37]. Nevertheless, many questions about IL-17 ligand-receptor relationships and IL-17 receptor signaling pathway are unanswered.

After activation of the IL-17RA by the binding of IL-17, IL-17 signaling has been shown to induce various downstream pathways which will be described below. IL-17 activates nuclear factor-κB (NF-κB) and mitogen-activated protein kinase (MAPK) pathways [38,39]. Schwander *et al *have found that tumor necrosis factor receptor-associated factor 6 is important for IL-17-induced NF-κB activation and the expression of IL-6 or intercellular adhesion molecule (ICAM)-1 [40]. Recent studies have shown that the adaptor protein NF-κB activator 1 (Act-1) plays an essential role in IL-17-dependent signaling [41-43]. The expression of inflammation-related genes induced by IL-17 is abolished in Act1-deficient cells [41,42]. However, an Act-1-independent signaling event such as activation of Janus kinase (JAK)1-associated phosphoinositide 3-kinase (PI3K) is described [44], and so the IL-17 signaling cascade is far from being completely defined.

IL-17 is known to induce the secretion of IL-6, CXCL8 (IL-8), granulocyte colony-stimulating factor (G-CSF), and prostaglandin E2 from normal synoviocytes and other adherent cells of various human tissues such as kidney epithelial cells, skin fibroblasts, brain endothelial cells, lung fibroblasts, and bronchial epithelial cells, thus indicating its link to inflammatory reaction [20,45]. When cultured in the presence of IL-17, fibroblasts sustained the proliferation of CD34^+ ^hematopoietic progenitors and their preferential maturation into neutrophils [45]. These results have suggested a potential contribution of IL-17 to neutrophil biology. Furthermore, a major group of IL-17 target genes is neutrophil-attracting chemokines, which include CXCL1 (Gro-α), CXCL2 (Gro-β), CXCL5, CXCL6 (GCP-2), CXCL8, and CCL2 (MCP-1) [45,46]. Therefore, the key biological function of IL-17 is associated with neutrophil-dominated inflammation, as a promoter of granulopoiesis, neutrophil accumulation, and neutrophil activation. In addition, IL-17 induces the expression of not only eosinophil-guiding chemokines like CCL5 (RANTES) and CCL11 (eotaxin) but also other inflammatory mediators like ICAM-1 and cyclooxygenase-2 [40,46-48]. A more recent study by Hsu *et al *has shown that blocking IL-17 signaling disrupts CD4^+ ^T cell and B cell interactions required for the formation of germinal centers and reduces humoral responses, indicating the significant impact of IL-17 on immune response [49]. Taken together, IL-17 acts as an orchestrating cytokine in immune and inflammatory responses.

### IL-17 in asthma

Since the discovery of IL-17 and its key property as a pro-inflammatory cytokine, IL-17 has been found to be closely associated with a range of inflammatory diseases, including rheumatoid arthritis, multiple sclerosis, inflammatory bowel diseases, and psoriatic disease [14,15]. Bronchial asthma is one of the IL-17-related diseases which have been studied actively.

#### 1) Involvement of IL-17 in asthma: clinical evidence

IL-17 is up-regulated in lung tissues, BAL fluids, sputum, and peripheral blood from patients with allergic asthma [16,17,50-55]. In the sputum of asthmatic patients, the increased levels of IL-17 mRNA expression correlate with the number of neutrophils [52]. Thus, IL-17 seems to contribute to neutrophilic accumulation in asthma, in accordance with the well-known biological function of IL-17 promoting neutrophil-dominated inflammation [51]. Although it has been well established that the pathognomonic features of asthma are mediated by eosinophils, mast cells, and Th2 cells [1], the number of neutrophils is increased in the airways of severe asthma [56]. Therefore, IL-17, whose signaling induces neutrophil recruitment into the airways, can be an important cytokine in the pathogenesis of asthma and the determination of the disease severity. Actually, the percentages of Th17 cells as well as the levels of IL-17 in airway and plasma tend to increase with the disease severity in asthmatic patients [17,55]. Somewhat surprisingly, the IL-17 mRNA levels correlate positively with the IL-5 mRNA levels in sputum from asthmatic patients [52]. These data may provide a potential clue regarding the association of IL-17 with Th2-mediated eosinophilic airway inflammation in asthma. Both in plasma and in activated peripheral blood mononuclear cells from allergic asthmatics, the increase in IL-17 concentration is accompanied by the enhanced concentration of IL-23 which is a critical regulator of IL-17 [55]. In addition, an increase in transcription factor RORγt level is found in allergic asthmatics [55]. These findings indicate that increased expression of IL-23 and RORγt may contribute to the increase in IL-17 expression in asthmatic patients. Therefore, the results in asthmatic patients suggest that besides predominant Th2 immunity, abnormal Th17 immunity is also involved in the pathogenesis of allergic asthma.

#### 2) Involvement of IL-17 in asthma: animal models and *in vitro *studies

In a murine model of allergen-induced airway inflammation, epicutaneous sensitization and subsequent inhalation challenge of ovalbumin (OVA) resulted in an increase in IL-17 expression of the lung, whereas intraperitoneal sensitization did not induce IL-17 response [57]. On the other hand, pulmonary IL-17 was induced in the mice sensitized subcutaneously with OVA [58]. Wilson *et al *have demonstrated that lipopolysaccharide (LPS) sensitization via the airway promotes strong Th17 responses with modest Th2 responses, while sensitization through the peritoneum primes strong Th2 responses [9]. In addition, after intranasal sensitization into the mice with a clinically relevant aeroallergen, house dust mite (HDM), IL-17 is produced in an antigen-specific manner [59,60]. These data have suggested that the method of sensitization or type of antigen may affect IL-17 response in murine model of allergic airway inflammation. Even so, most of previous studies using mouse models of asthma have shown that IL-17 expression in the airways is up-regulated after sensitization and challenge with the antigen, agreeing with the human data [11,13,61]. All of these observations suggest that IL-17 is involved in the pathogenesis of allergic asthma.

As research on the role of IL-17 in allergic airway inflammation is still in an early stage, there is limited literature addressing the regulation of IL-17 and its related molecules in asthma. The main source of IL-17, Th17 cells have been suggested to migrate into the asthmatic airway following antigen challenge in mouse models of asthma [4,9,62]. In addition, natural killer T cells and γδ T cells, which produce IL-17 in the lung, contribute to the asthmatic responses through secretion of Th2 cytokines [9,62,63]. Alveolar macrophages also produce IL-17 and promote asthmatic development in OVA-inhaled mice [64]. Regarding the role of IL-23 in allergic airway inflammation, a previous study has reported that IL-23 level is increased in the lung upon OVA challenge in a murine model of asthma [58]. In cells from mediastinal lymph nodes of OVA-sensitized and -challenged mice, IL-23 is able to induce IL-17 production, indicating a significant contribution of IL-23 to IL-17 induction in OVA-induced asthma. The production of IL-17 after challenge of LPS-contaminated allergens is blunted in IL-6 knockout mice, suggesting that IL-6 production by LPS is critical for the development of allergen-specific Th17 polarization [61]. Krishnamoorthy *et al *have demonstrated a central role of c-Kit expressed by dendritic cells in the fine-tuning of the IL-6 expression, which promotes both Th17 and Th2 responses upon stimulation of HDM [59]. The other study has shown that the Toll/IL-1 receptor adaptor protein, MyD88 is responsible for the development of Th17-mediated allergic airway inflammation in response to HDM [60]. These observations have suggested that allergen-induced IL-17 responses in asthma are regulated via a complex network involving several molecules and various cell types.

A specific role of IL-17 in asthma becomes a matter of primary scientific concern. Supporting the observations in asthmatic patients regarding the role of IL-17 in neutrophilic inflammation, intratracheal administration of IL-17 increases the absolute number of neutrophils in BAL fluids of rat [63]. IL-17 per se, however, does not cause chemotaxis of human neutrophils from peripheral blood, when studied *in vitro *[64]. In contrast, IL-17 enhances the production of IL-8 in human airway smooth muscle cells, bronchial epithelial cells, and bronchial fibroblast, and the neutrophil chemotactic effect of IL-17 is blocked by an anti-IL-8 antibody (Ab) *in vitro *[16,64]. These data indicate that IL-17 exerts the accumulation of neutrophils into the airways in an indirect manner, mainly via the enhanced production of IL-8, a potent neutrophilic chemoattractant by lung structural cells [64,65]. Consistent with previous *in vitro *studies, our recent study with a murine model of asthma has shown that inhibition of IL-17 activity with an anti-IL-17 Ab remarkably reduces the increase in airway infiltration of neutrophils and expression of KC (a functional murine homolog of IL-8) protein and mRNA induced by allergen inhalation [66]. Moreover, IL-17 augments the release of IL-6 from human bronchial fibroblasts and expression of G-CSF in bronchial epithelial cells, inducing neutrophil development and granulopoiesis [16,33]. Thus, IL-17 in asthma orchestrates neutrophilic airway inflammation by inducing the release of neutrophilic chemoattractants and activating factors from local cells in the lungs.

While the contribution of IL-17 to neutrophilic asthma has been consistently reported, the direct regulatory effect and mechanism of IL-17 on eosinophilic airway inflammation are somewhat complex to decipher. Interestingly, Schnyder-Candrian *et al *have reported that IL-17 receptor gene-deficient mice show a reduced recruitment of neutrophils as well as eosinophils into the airway after antigen challenge [58]. Furthermore, activity of eosinophil peroxidase in lung tissues and serum concentrations of OVA-specific serum IgE are also reduced in the IL-17 receptor deficient mice [58]. In addition, an involvement of IL-17 in the activation of allergen-specific T cells has been demonstrated using IL-17-deficient mice [67]. Wakashin and colleagues have reported that adoptive transfer of antigen-specific Th17 cells to mice significantly enhances antigen-induced, Th2 cell-mediated recruitment of eosinophils into the airways and AHR [8]. Supporting the role of IL-17 in Th2 cell-mediated and eosinophilic inflammation in asthma, our recent study has shown that inhibition of IL-17 activity with anti-IL-17 Ab reduces remarkably allergen-induced airway infiltration of inflammatory cells including eosinophils, Th2 cytokine levels in BAL fluids, and AHR [68]. On the other hand, other study has reported that neutralization of IL-17 augments the allergic responses in sensitized mice and that intranasal co-administration of OVA with IL-17 reduces pulmonary eosinophil recruitment and AHR, proposing a novel regulatory role of IL-17 [58]. IL-17 seems to decrease CCL11 expression and reduce thymus- and activation-regulated chemokine/CCL17 (TARC) production in dendritic cells upon antigen restimulation. These results have suggested that IL-17 has a dual role in the regulation of eosinophilic airway inflammation in asthma. Thus, IL-17 promotes eosinophilic airway inflammation by mounting Th2 responses during antigen sensitization while inhibiting eosinophilic airway inflammation by acting as a down-regulator of the dendritic cell-derived Th2 chemoattractant TARC during the effector phase [8,58]. However, more recent studies have reported that administration of anti-IL-17 Ab to OVA-inhaled mice in the challenge phase reduces antigen-induced airway infiltration of eosinophils and Th2 cytokine levels in BAL fluids by using different sensitization and challenge protocols [13,68]. In addition, an enhancing effect of IL-17 on CCL11 mRNA expression and protein release in human airway smooth muscle cells has been reported [47,69]. Moreover, IL-17 activates NF-κB pathway [38,39] that can subsequently induce CCL11 and TARC expression, suspecting the presence of an indirect regulatory pathway [47,70]. These observations suggest that IL-17 is associated with Th2 cell-mediated eosinophilic inflammation in asthma, although a few controversial issues and the direct regulatory mechanism remain to be clarified. The discrepancy in the results obtained from various animal models can be explained by different experimental conditions, such as protocols and routes of sensitization and challenge, types of antigens, and mouse strain. This variability may be relevant to humans. Furthermore, asthma is a complex, heterogeneous disease that cannot be explained by one underlying mechanism [71]. Therefore, the mode or level of IL-17's contribution to allergic inflammation may be different depending on asthma endotypes. Taken together, even though more studies are required to determine the primary role and downstream signaling mechanism of IL-17, IL-17 seems to contribute to neutrophilic inflammation as well as Th2 cell-mediated and eosinophilic inflammation in asthma.

Mucus hypersecretion and persistent airway inflammation are common features of asthma. Chen *et al *have found that IL-17 stimulates the expression of mucin genes, MUC5B and MUC5AC, in the airway epithelial cells and that its effect on MUC5B expression is at least partly mediated by IL-6 in a JAK2-dependent pathway [72]. As the contribution of excessive airway mucus to asthma pathology is clear, the increase in mucus production can be an action mechanism of IL-17 in asthma. The potential significance of Th17 responses in asthma stems partly from the effects of Th17 cells on the function of regulatory T cells controlling effector T cell responses [73]. Thus, the inhibition of IL-17 can be a way to control inflammation but also to restore regulatory T cell functions in asthma [74]. In short, accumulating evidence indicates that IL-17 is implicated to the pathogenesis of asthma, suggesting IL-17 as an attractive therapeutic target for asthma.

#### 3) Limitations of targeting IL-17 in asthma

Although the information regarding direct involvement of IL-17 in human defense mechanism is still limited, studies using animal models of infection provide evidence for the role of IL-17 in mammalian immune response against pathogens [14]. IL-17 contributes to host defense against respiratory bacteria and fungi through its promoting effects on neutrophil recruitment and activation [65,75]. In addition, IL-17 up-regulates several acute phase response proteins and antimicrobial proteins, including serum amyloid A, C-reactive protein, lipocalin 2, β-defensins, and S100 proteins [48,75]. Actually, mice lacking either IL-17 or IL-17RA are susceptible to lung infection with the gram negative bacteria [65,75]. STAT3 plays a critical role in Th17 development and thus displays multiple functions in host defense against extracellular pathogens. Recent studies have demonstrated that mutations in STAT3 in animal models and humans confer a defect in IL-17 function and result in high susceptibility to respiratory infections with extracellular bacteria and fungi [75-77]. These findings indicate that blockade of IL-17 may be associated with an increased risk of opportunistic infections. In addition, IL-17 is involved in regulation of tumor immunity [78]. A major function of IL-17 in the tumor microenvironment is to stimulate tumor growth and progression by facilitating angiogenesis [79]. On the other hand, IL-17 may suppress tumor cell growth through promoting an antitumor cytotoxic T cell response [80]. Therefore, for the development of IL-17-targeting agents to treat human diseases, researchers should keep in mind that inhibition of IL-17 responses bears potential risk for impairing patients' host defense or antitumor activity.

### Targeting IL-17 for treatment of asthma

**Figure 1:**
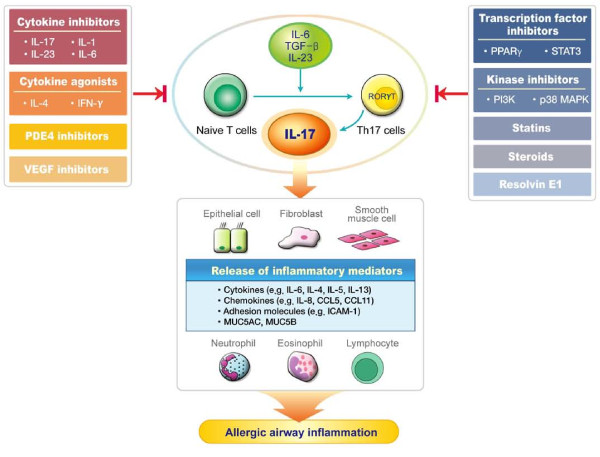
**Potential strategies to regulate the IL-17 pathway for the treatment of asthma**. T-helper 17 (Th17) cells are differentiated under the control of interleukin (IL)-6, transforming growth factor (TGF)-β, and IL-23. IL-17 produced predominantly by Th17 cells augments allergic airway inflammation by inducing the expression of various pro-inflammatory mediators such as cytokines, chemokines, and adhesion molecules, in turn leading to recruitment and activation of neutrophils and Th2-mediated eosinophils. The modulation of cytokines or transcription factors, inhibition of kinases, phosphodiesterase 4 (PDE4), vascular endothelial growth factor (VEGF), and pharmacological agents such as statins, steroids, and resolvin E1 down-regulate IL-17 expression, thus ameliorating allergic airway inflammation.

Various strategies to down-modulate the IL-17 responses by inhibiting upstream or downstream molecules involved in IL-17 signaling and by blocking IL-17 per se as well as by regulating the differentiation and activation of Th17 cells have been applied as a therapeutic approach for many inflammatory diseases [14]. Here, we discuss the up-to-date modalities to inhibit IL-17 responses with therapeutic effects on asthma (Figure 1).

#### 1) Blockade of IL-17 activity de novo

The blockade of IL-17 activity with anti-IL-17 Ab decreases the numbers of total cells lymphocytes, neutrophils, and eosinophils in BAL fluids increased after antigen challenge as compared with the numbers in allergen-inhaled mice administered isotype control monoclonal Ab [18,71]. Treatment with the anti-IL-17 Ab also reduces the levels of IL-4, IL-5, and IL-13 in BAL fluids and AHR [71]. Inspiringly, the clinical trials evaluating the efficacy, safety, and tolerability of a monoclonal Ab against IL-17 in treatment-resistant patients with various inflammatory diseases such as Crohn's disease, rheumatoid arthritis, and psoriasis are underway [28,81]. Therefore, the favorable data from animal models of allergen-induced airway inflammatory disease can provide us with the rationale for investigating therapeutic effects of anti-IL-17 Ab in asthmatic patients.

Other possible targets to inhibit IL-17 action include receptors for IL-17, IL-17RA and IL-17RC. Human bronchial epithelial cells pretreated with anti-IL-17R Ab show a decrease in IL-17 activity [82]. Kuestner and colleagues have produced a soluble form of IL-17RC, which binds to IL-17 and IL-17F with high affinity and thereby inhibits the signaling of these cytokines in fibroblast [36]. In addition, cultured splenocytes from IL-17RA knockout mice, unlike wild-type mice, did not produce IL-5 or IL-13 by the stimulation with IL-25 which is known to regulate allergen-induced Th2 responses and AHR [83,84]. Furthermore, treatment with a monoclonal IL-17RA Ab completely inhibited IL-25-induced pulmonary inflammation and AHR [84]. Based on these data, the effect of targeting IL-17 receptor in allergen-induced airway inflammation is anticipated.

#### 2) Modulation of cytokines regulating IL-17 production

As IL-23 is required for full acquisition of pathogenic function of Th17 cells including IL-17 production, blocking IL-23 may be a promising therapeutic approach to reduce IL-17 production [8,30]. IL-23 produced by antigen-presenting cells such as macrophages and dendrite cells induces IL-17 production, which then stimulates the recruitment of inflammatory cells into the lung in murine models of asthma [58,85,86]. In accordance with these animal studies, a parallel elevation of IL-17 and IL-23 concentrations in allergic asthmatic patients is observed, suggesting that IL-23 functions as an important regulatory cytokine involved in the Th17-induced inflammation of allergic asthma [55]. A recent study has shown that the increased IL-23 expression in the airways in lung-specific IL-23 transgenic mice is associated with antigen-induced Th2 cytokine production, eosinophil accumulation in the airways, goblet cell hyperplasia, and AHR [8]. Moreover, neutralization of IL-23 activity with an Ab against IL-23 p19, significantly inhibits the antigen-induced recruitment of lymphocytes, eosinophils, and neutrophils into the airways and decreases the production of Th2 cytokines in the airways of OVA-sensitized mice [8]. These results have provided a possibility that IL-23 inhibition can be a therapeutic strategy reducing IL-17 expression in asthma. For other inflammatory diseases that IL-23 is implicated their pathogenesis, the preclinical and clinical studies with some IL-23 inhibitors have been undertaken [14]. For an example, promising results have been obtained from clinical studies with an oral IL-12/IL-23 inhibitor, STA-5324, in Crohn's disease [87]. Additionally, ustekinumab (CNTO-1275), a subcantaneous monoclonal Ab against the p40 subunit of IL-12 and IL-23 was developed to treat several inflammatory diseases such as psoriasis, psoriatic arthritis, multiple sclerosis, and Crohn's disease [88]. Very recently, a randomised, double-blind, placebo-controlled, crossover trial has shown the efficacy and safety of ustekinumab in treating psoriatic arthritis [89]. The positive results obtained from these clinical studies encourage us to apply to other IL-23/IL-17-related diseases, including asthma.

IL-6 is known as a strong inducer of Th17 cells [26]. Targeting the IL-6 receptor with a monoclonal Ab was shown to be an effective approach for treating systemic-onset juvenile idiopathic arthritis [90]. However, McGeachy *et al *have reported that IL-6 with TGF-β can drive the production of IL-17 but restrain the pathogenic potential of Th17 cells through up-regulating the production of IL-10, a regulatory cytokine [91]. More recently, a reciprocal role of IL-6 in generation of Th17 cells and regulatory T cells in immune response has been proposed [92]. IL-6 signaling inhibits the conversion of conventional T cells into Foxp3^+ ^regulatory T cells *in vivo *[93]. These data have suggested that IL-6 is a potent regulatory factor switching immune responses from the induction of Foxp3^+ ^regulatory T cells to pathogenic Th17 cells. Therefore, clarifying the effect of IL-6 in balancing between functions of Th17 cells and regulatory T cells during allergic responses will make it more clear that blockade of IL-6 is effective for the inhibition of Th17 cell-mediated inflammation in asthma.

IL-1 plays a pivotal role in developing Th17 cells and eliciting inflammation [94]. IL-1 deficiency or administration of anti-IL-1 receptor type I Ab significantly suppresses the development of arthritis in mice [95]. Interestingly, treatment with a commercially available recombinant IL-1 receptor antagonist (anakinra) results in clinical response in nine patients with systemic onset juvenile idiopathic arthritis that was resistant to conventional aggressive treatment [96]. A previous study has reported that recombinant human IL-1 receptor antagonist effectively suppresses allergen-induced asthmatic symptoms in animal models [97]. However, it is not clear whether the therapeutic effect of the IL-1 receptor antagonist in asthma is due to modulation of Th17 cell development and IL-17 production.

IL-4 and IFN-γ negatively regulate the generation and population expansion of IL-17- producing T cells and their expression of IL-17, which may serve as a protective strategy to fine-tune the expression of IL-17 [5,6]. In OVA-sensitized and -challenged mice, *in vivo *IFN-γ gene delivery through the intravascular injection of plasmid DNA has been shown to suppress airway eosinophilia, IL-5 and IL-13 production, and bronchial mucus production with reducing the production of IL-17 and IFN-γ itself in the lung [98]. These results suggest that IFN-γ has a broad immune regulatory potential including the suppression of IL-17 production in the lung.

#### 3) Transcription factor inhibitors

Peroxisome proliferator-activated receptors (PPARs) are members of the nuclear receptor superfamily that regulate gene expression [99]. Among three PPAR subtypes, PPARγ activation down-regulates the synthesis and release of immunomodulatory cytokines from various inflammatory cells [100]. Our recent study has shown that administration of PPARγ agonists, rosiglitazone and pioglitazone decreases the IL-17 protein and mRNA expression in the lung and reduces Th2 cytokine expression, AHR, and eosinophil activation, which are increased by induction of asthma [68]. In addition, the attenuating effect of PPARγ agonist on allergic airway inflammation and AHR is abrogated by exogenous IL-17 administration. Accordingly, these results suggest that the therapeutic effect of PPARγ agonist in asthma is exerted by the down-regulation of IL-17 expression, providing a piece of evidence for an interaction between PPARγ signaling and IL-17 expression in allergic airway inflammation.

STAT3 is a transcriptional activator required for IL-17 responses. Saleh *et al *have shown that IL-17-mediated CC chemokine (CCL11) promoter activity and mRNA expression are decreased in STAT3-silenced airway smooth muscle cells, demonstrating the possible role of IL-17/STAT3 signaling pathway in airway inflammatory responses [69].

#### 4) Kinase inhibitors

PI3K phosphorylates phosphatidylinositol 4,5-bisphosphate, forming a lipid second messenger, phosphatidylinositol 3,4,5-trisphosphate that controls a variety of intracellular signaling pathways. PI3K is negatively regulated by phosphatase and tensin homolog deleted on chromosome 10 (PTEN). In a murine model of toluene diisocyanate-induced asthma, administration of PI3K inhibitors or gene transfer of PTEN reduces the increase in IL-17 expression in the lung with attenuation of allergen-induced airway inflammation and AHR [13]. In addition, our recent data have revealed that selective inhibition of PI3Kδ isoform remarkably reduces IL-17 expression induced by an allergen challenge [66]. Thus, PI3K/PTEN pathway, especially the PI3Kδ pathway, seems to be a signaling system that regulates IL-17 expression in allergic airway inflammation.

IL-17-induced release of IL-6 and IL-8 in bronchial epithelial cells is inhibited by the inhibitor of p38 MAPK, SB202190 and by the inhibitor of extracellular signal regulated kinase (ERK), PD98059 in a concentration-dependent manner [101]. The inhibition of p38 MAPK attenuates the release of CXCL1 and CXCL6 induced by IL-17, while blocking the ERK signaling does not display any substantial effect on the release of these chemokines [102]. These observations indicate that p38 MAPK signaling pathway may be the potential pharmacotherapeutical target for the IL-17-mediated airway neutrophilic inflammation.

#### 5) Phosphodiesterase 4 (PDE4) inhibitors

Selective PDE4 inhibitors, which elevate intracellular cAMP by inhibiting the hydrolysis of cAMP, are effective anti-inflammatory agents in airway inflammatory diseases. A recent study using peripheral blood mononuclear cells and purified CD4^+ ^T cells has demonstrated that treatment of a selective PDE4 inhibitor Zl-n-91 suppresses IL-17 production [103]. Consistent with *in vitro *results, a selective PDE4 inhibitor, roflumilast decreases the expression of IL-17 mRNA in the airways and suppresses both subepithelial fibrosis and airway epithelial hypertrophy induced by chronic challenge with OVA in mice [104]. These results suggest that PDE4 inhibitors have beneficial effects, at least in part by inhibiting IL-17 production, in allergen-induced airway inflammation and airway remodeling in asthma.

#### 6) Vascular endothelial growth factor (VEGF) inhibitors

VEGF, an endothelial cell-specific mitogenic peptide, is a well-known potent promoter of vasculogenesis and angiogenesis. Also, VEGF enhances microvascular permeability, thereby inducing the migration of inflammatory cells to the airway [105]. Therefore, VEGF has been recognized as a crucial stimulator of airway inflammation, AHR, airway remodeling, and Th2 immune responses in asthma. Very recently, the role of VEGF in the polarization to Th17 cells in a murine model of asthma induced by airway sensitization with LPS-contaminated allergens has been evaluated [61]. Treatment with a VEGF receptor inhibitor, SU5416, decreased significantly the levels of IL-17 in BAL fluids after allergen challenge and this effect was accompanied by inhibition of the production of a Th17 polarizing cytokine, IL-6. In addition, the blockade of VEGF signaling by a cyclopeptidic vascular endothelial growth inhibitor, CBO-P11 and a novel VEGF blocker, VEGF-Trap also reduces the IL-17 levels increased after OVA inhalation in a mouse model of asthma (our unpublished data). Therefore, inhibition of VEGF activity is suggested to ameliorate airway inflammation and AHR, regulating the polarization to Th17 cells and the production of IL-17 in asthma.

#### 7) Statins

Statins are widely used to manage the patients with hyperlipidemia. Interestingly, these cholesterol-lowering agents have been shown to exhibit immunosuppressive effect in several immune-mediated disease models [106]. Simvastatin attenuates release of airway neutrophilic and remodeling mediators and inhibits their up-regulation induced by IL-17 in primary bronchial epithelial cells [107]. Yeh and Huang have reported that treatment of mice with pravastatin reduces airway eosinophilia increased after inhalation of OVA [108]. Moreover, administration of pravastatin suppressed OVA-induced proliferation and production of Th2 cytokines in spleen cells *ex vivo *and *in vitro *[108]. In addition, pravastatin suppressed IL-17 production in the thoracic lymph node, eosinophilic airway inflammation, and OVA-specific IgE production in a murine model of asthma [106]. These results indicate that statins down-regulate IL-17 production and thus suppress allergic responses in the airway, suggesting that statins can be a new therapeutic option targeting IL-17 for asthma.

#### 8) Steroids

Among the current treatments for asthma, steroids are the most commonly used controller as a potent anti-inflammatory agent. The IL-17-induced release of IL-8, CXCL1, and CXCL6 from human bronchial epithelial cells might be sensitive to glucocorticoid receptor stimulation [102]. In contrast, another study has reported that dexamethasone fails to attenuate the IL-17-induced release of IL-8 in human airway smooth muscle cells [109]. In animal model of allergic airway inflammation, treatment with dexamethasone significantly decreased mRNA expression of several cytokines including IL-17, which are increased in the airways after chronic challenge with OVA [104]. A recent study has also shown that dexamethasone can inhibit the release of IL-17 by inhibiting RORγt expression and blocking Th17 differentiation in a murine model of OVA-induced asthma [110]. On the contrary, McKinley *et al *have demonstrated that Th17 cell-mediated airway inflammation and AHR are steroid resistant in mice, suggesting a potential implication of Th17 cells in steroid-resistant asthma [111]. Therefore, whether steroids exert their therapeutic effect through regulating IL-17 production in asthma remains to be evaluated.

#### 9) Resolvin E1

Epidemiological studies have found that diets rich in omega-3 fatty acids lower the prevalence of asthma [112]. Resolvins are products of omega-3 fatty acids named on the basis of their original identification in resolving exudates and their ability to exert potent anti-inflammatory properties, accelerating the resolution phase of acute inflammation [113]. In a mouse model of OVA-induced allergic airway inflammation, resolvin E1 promotes the resolution of the inflammatory airway responses in part by directly suppressing the production of IL-23 and IL-6 in the lung [114]. Thus, administration of resolvin E1 decreases concentration of IL-17 protein in BAL fluids and a decreased ratio of IL-17-producing to IFNγ-producing T cells [114]. The regulation of adaptive immune responses by resolvin E1 provides a therapeutic strategy via IL-17 regulation for the persistent and unrestrained immune responses of asthma.

## Conclusion

Cytokines play a key role in orchestrating airway inflammation and structural changes of the respiratory tract in asthma, and thus become an important target for the development of therapeutic modalities for the disease. Therefore, the discovery of new cytokine can afford other opportunity to control the inflammatory diseases. Recent studies have provided convincing evidence that IL-17, the predominant product of Th17 cells, plays an imperative role in regulating the expression of inflammatory mediators and the recruitment and function of inflammatory cells in various inflammatory diseases including asthma. As the regulatory systems involved in the differentiation of Th17 cells and the production of IL-17 have been identified, this knowledge has allowed the rationale for the development of novel therapeutic agents targeting IL-17 in asthma. It is very encouraging that the clinical trials on an anti-IL-17 Ab in the patients with IL-17-associated inflammatory diseases are underway, although not in asthmatic patients. With regard to asthma, studies using animal models of asthma are on the way, and the data supporting the therapeutic potential of strategies inhibiting IL-17 expression on allergic airway inflammation and AHR are accumulating. In the near future, the question on whether IL-17 is a reliable therapeutic target for asthmatic patients will be answered, hopefully.

## Competing interests

The authors declare that they have no competing interests.

## Authors' contributions

SJP and YCL contributed to conception and design and drafted the manuscript. All authors read and approved the final manuscript.
